# How to approach patients after Stevens-Johnson syndrome with multiple drug involvement: test or not to test? To attest allergy to all involved drugs without testing?

**DOI:** 10.1186/2045-7022-4-S3-P98

**Published:** 2014-07-18

**Authors:** Tamar Kinaciyan

**Affiliations:** 1 Medical University of Vienna, DIAID, Dept of Dermatology, Austria

## Background

A 36 years-old woman who suffered from Stevens-Johnson Syndrome (SJS) 9 months ago is reported. She was hospitalized for pneumonia with fever (39°C), cough and severe pain after 5 days of oral amoxicillin (AMX) and clavulanic acid treatment. Therapy was changed to cefuroxime i.v. and clarithromycin and acetylcysteine p.o. and diclofenac novalgin or perfalgan i.v. as needed. Two days later her general condition worsened, therefore therapy regimen was changed to ampicillin. She further developed conjunctivitis and later on erosive stomatitis. Two more days later coin-sized lesions arised on the back and rapidly generalized. The dermatology consultant diagnosed a SJS and took over the patient. All ongoing treatments were stopped, high dose, corticosteroids iv. and supportive treatment started. Bacterial and viral tests revealed a mycoplasma pneumonia which then was treated with doxycycline. After 3 weeks, she could be discharged and got an allergy passport for amoxicillin, clavulanic acid, paracetamol, NSAIDs, paracodein and due to possible cross-senstivity, all beta-lactams should be avoided.

## Methods

allergologic work-up was performed with i.d. tests (reading after 24 h), patch tests de loco with all involved drugs and some alternatives for NSAIDs. Drug provocation tests (DPT) with lornoxicam as an alternative NSAID and penicillin V as a representative for beta-lactams were carried out carefully and gradually over days.

## Results

All skin tests resulted negative. DPT with lornoxicam and penicillin V were well tolerated without any side effects.

## Conclusions

Patients with such a case history and the described allergy passport will hardly find a physician to treat her. In case of analgesics, only morphine derivatives and in case of antibiotics only few alternatives can be prescribed. Therefore, a careful diagnostic evaluation is required even if currently it is in general recommended not to test these patients. Moreover, in this case, the mycoplasma infection may also have triggered the SJS. We could make out for the patient at least an alternative NSAID for oral and i.v. application and dissolve the generous ban for all beta-lactams.

